# Treatment of Otorrhœa

**Published:** 1891-08-15

**Authors:** 


### OTOLOGY.

### TREATMENT OF OTORRHŒA.

The discharge may come from the meatus, or it may come from the middle ear, as in otitis media.

In the former class of cases, it may result from any acute inflammatory condition of the meatus. In children, furuncle of the meatus, otitis externa circumscripta, that is, a limited inflammation in the subcutaneous areolar tissue, ending in the formation of an abscess, is often seen. The suppurating point may discharge, and all trouble cease, but not infrequently a chronic discharge is left. It is very desirable that, if possible, the inflammation should be treated in the earlier stages. We have usually found that a few drops of tincture of opium in the external meatus, and the administration of small doses of tincture of pulsatilla have been followed by relief. Professor Grüber states that since he introduced into aural practice the use of medicated gelatine preparations, he almost always begins the treatment with these. In the early stage, in which severe pain is usually present, he employs "amygdalæ aurium," containing one-sixth of a grain of the liquid extract of opium, or one-twelfth of a grain of hydrochlorate of morphine. The auditory canal is first thoroughly washed out with a warm four per cent. solution of carbolic acid; the gelatine-almond is then introduced deeply into the canal with the aural forceps, and the meatus closed with cotton wool. The preparation gradually dissolves, and the pain is in this way generally very soon alleviated, and the inflammatory process cut short in many patients. The applications may be repeated as needed; sometimes one daily is sufficient, in other cases three or four may be indicated. In children, half may be used.

The effects of these preparations are stated by the learned aurist to be two-fold—partly anæsthetic and partly antiphlogistic—and he strongly recommends them on account of their value in appropriate cases, and from the simplicity of the treatment. If pus has already formed, their advantage is not so marked, though they are still very useful for the relief of pain until the abscess is evacuated.

But the furuncles not infrequently depend on the presence of certain micro-organisms, and as an abortive measure, with the object of destroying the micro-organisms associated with the furuncular inflammation, Häter and Weber-Liel recommend the subcutaneous injection of a few drops (two to four) of a five per cent. solution of carbolic acid. The pain abates in about a quarter of an hour; and he advises instillations of spirit to be then employed in about three hours' time, and repeated every two hours.

A diffuse inflammation of the external auditory canal is another cause of chronic purulent discharge from the meatus.

Otorrhœa is a term signifying a flowing or running from the ear, the meatus externus, and should therefore be applied to a symptom rather than a distinct disease. The practical importance of duly recognising the varieties of otorrhœa has been duly insisted on by Dr. Swift Walker*, who takes exception to the use of the word "otorrhœa," simple inflammations of the external auditory meatus, for, as a matter of fact, the disease only becomes "otorrhœa" when it has become "chronic" by perforation of the tympanic membrane, each variety requiring its special treatment. In consequence of care not being taken to diagnose the characteristic variety of the disease its treatment becomes a matter of routine, and, in many cases, inefficacious; thus the disease has become an opprobrium to most practitioners. The simplest form arises from dentition and stomach derangement in children; but these cases are generally neglected, under the impression that the discharge will cease without remedial measures, a most delusive impression, and one that it is the duty of every practitioner to remove, as if allowed to go on it is certain to

* *Lancet*, Nov. 1, 1890.

destroy the tympanum or cause perforation. If a disinfectant is used, the author prefers resorcin for the ear, and is of opinion that carbolic acid, which is his general favourite, is most irritating to an inflamed meatus and tympanum. When the case becomes chronic, or an old neglected sore comes under notice, it becomes a question whether the dry plan of treatment is to be preferred; if so, the following mode should be adopted: Take an ear probe, and having inserted a small piece of cotton wool in the meatus, press it down with the flat end of the probe, then remove the plug. If Messrs. Krohne and Seseman's probe be used, the corkscrew at one end removes the wool without difficulty. The method clears the meatus of all discharge without any pain, which is a great desideratum. The first day disinfect the meatus with a little iodoform, inflated by Kabinsky's inflator; use on the second and following days with the following powder: Talc (creta Gallica), three drachms; boric acid, one drachm. This Dr. Walker finds is a very useful powder, and has been used by him for years. If it does not succeed at the end of a week he uses creoline dusting powder. In the perforative variety of otorrhœa the same precautions are necessary in cleansing the meatus daily, but they cannot be treated on the dry plan. The author gives the following as a good lotion: Resorcin, half a scruple; extract of opium, one drachm; chloride of zinc, one grain; water to one ounce and a half. A little to be syringed into the ear each morning. The beneficial results obtained by the use of a one per cent. solution of styrone in such cases was mentioned in the article on this new disinfectant in The Hospital for June 27, page 154.

Many diseases are liable to be complicated with otitis media, ending in suppuration, and perforation of the tympanum; among these are measles, scarlet fever and small-pox, tubercle, syphilis, typhoid fever, recurrent fever, ulcerative endocarditis.

We have no reason to dispute Grüber's statement that amongst substances useful in otorrhœa finely-powdered boric acid takes the first place (a remedy first introduced for this purpose by Bezold). The auditory canal is washed out with a boric acid solution, and all foreign matters removed as far as possible from the middle ear into the air ball. The canal is then dried properly, a little of the powdered boric acid is blown in, and the canal plugged with cotton wool.

Dr. Walker, in his paper, to which we have fully referred above, finds that a very good lotion is alum iron, three drachms; sulphate of zinc, two drachms; water to eight ounces, the *modus operandi* being the same as for the boric acid insufflation.

